# μ-band desynchronization in the contralateral central and central-parietal areas predicts proprioceptive acuity

**DOI:** 10.3389/fnhum.2023.1000832

**Published:** 2023-03-15

**Authors:** Giulia Aurora Albanese, Francesca Marini, Pietro Morasso, Claudio Campus, Jacopo Zenzeri

**Affiliations:** ^1^Department of Robotics, Brain and Cognitive Sciences, Fondazione Istituto Italiano di Tecnologia, Genoa, Italy; ^2^Department of Informatics, Bioengineering, Robotics and Systems Engineering (DIBRIS), University of Genoa, Genoa, Italy; ^3^Microsoft, Milan, Italy; ^4^U-VIP Unit for Visually Impaired People, Istituto Italiano di Tecnologia, Genoa, Italy; ^5^ReWing S.r.l., Milan, Italy

**Keywords:** proprioception, EEG, robotics, upper limb, neural correlates, position sense

## Abstract

**Introduction:**

Position sense, which belongs to the sensory stream called proprioception, is pivotal for proper movement execution. Its comprehensive understanding is needed to fill existing knowledge gaps in human physiology, motor control, neurorehabilitation, and prosthetics. Although numerous studies have focused on different aspects of proprioception in humans, what has not been fully investigated so far are the neural correlates of proprioceptive acuity at the joints.

**Methods:**

Here, we implemented a robot-based position sense test to elucidate the correlation between patterns of neural activity and the degree of accuracy and precision exhibited by the subjects. Eighteen healthy participants performed the test, and their electroencephalographic (EEG) activity was analyzed in its μ band (8–12 Hz), as the frequency band related to voluntary movement and somatosensory stimulation.

**Results:**

We observed a significant positive correlation between the matching error, representing proprioceptive acuity, and the strength of the activation in contralateral hand motor and sensorimotor areas (left central and central-parietal areas). In absence of visual feedback, these same regions of interest (ROIs) presented a higher activation level compared to the association and visual areas. Remarkably, central and central-parietal activation was still observed when visual feedback was added, although a consistent activation in association and visual areas came up.

**Conclusion:**

Summing up, this study supports the existence of a specific link between the magnitude of activation of motor and sensorimotor areas related to upper limb proprioceptive processing and the proprioceptive acuity at the joints.

## 1. Introduction

Proprioception is the sensory mechanism that allows humans to perceive body position and movement (Proske and Gandevia, 2012). It is essential for motor control and its decline due to neurological disorders or orthopedic injuries (Albanese et al., 2021b) can severely affect the human motor functions. Analysis of movements performed without vision by patients with large-fiber sensory neuropathy have demonstrated higher variability of limb position or force exerted than control subjects (Rothwell et al., 1982; Sanes et al., 1985; Forget and Lamarre, 1987; Ghez et al., 1990). Increased movement variability results from an inability to detect errors made during motion and the consequent failure to apply internal feedback mechanisms. Proprioception is a critical source of information in the promotion of task-specific neural development (Xerri et al., 1998; Schwenkreis et al., 2001; Pleger et al., 2003; Xerri, 2012) and its deeper investigation may allow for finding efficient methods to promote neuroplasticity and processes of brain recovery (Schabrun and Hillier, 2009). The proprioceptive mechanisms underlying human movement control are still under investigation and it remains unclear how proprioception is integrated and processed to control the dynamic interaction among limb segments (Ghez and Sainburg, 1995).

Previous studies have characterized human proprioception from a wide variety of aspects. From a physiological perspective, outstanding definitions of proprioception and its receptors have been provided to explain crucial basic mechanism (Proske and Gandevia, 2009, 2012; Hagert, 2010). Most research was focused on the assessment of joint proprioception, which is also called position sense. A comprehensive characterization of human position sense has been reached for the shoulder (Lephart et al., 1994), elbow (Soechting, 1982), knee (Skinner and Barrack, 1991), wrist (Albanese et al., 2021a), and ankle (Lephart et al., 1998) joints. Moreover, position sense acuity has been characterized in relation to the difference between left and right body sides (Goble and Brown, 2010), its development and deterioration with age (Skinner et al., 1984; Marini et al., 2017b), the influence of external forces (Kuling et al., 2013; Marini et al., 2017a), and the effects related to muscle fatigue (Albanese et al., 2022).

Despite this remarkable amount of work, what has been poorly investigated so far is to what extent brain activation during a proprioceptive task is associated with acuity at the joints. Past studies with functional magnetic resonance (fMRI) investigated the brain mechanisms of the feeling of ownership of seen body parts (Ehrsson et al., 2004). Researchers obtained the first brain map of neural activity related to proprioception at the ankle by stimulating the muscle spindles [key receptors for proprioception (Proske, 2006)] in the feet through tendon vibration (Goble et al., 2011). These studies employed vibrotactile stimulation to elicit illusory movements and provided a map of brain activation due to limb proprioception (Goble et al., 2011, 2012; Naito et al., 2016). Specifically, in this map, the areas of the brain in which muscle spindle-related neural activity was identified were the basal ganglia, the primary and secondary sensorimotor cortices, and the secondary association areas. Unfortunately, the limit of such fMRI studies, which investigate the neural basis of proprioception, lies in the impossibility of disjoining and detaching proprioception from motion (Scott, 2012), thus constraining the study of position sense in the absence of actual movement. Though such findings provided interesting insights into the neural basis of proprioceptive processing, the absence of actual limb motion did not allow us to fully and reliably extend them to real limb position sense (Han et al., 2016; Kenzie et al., 2018). Other studies with electrophysiological measures characterized the cortical representation of motion, but only in passive movements (Alary et al., 1998; Alegre et al., 2002; Seiss et al., 2002), or aimed at identifying differences in brain activation between disabled individuals and healthy subjects, with no consideration of factors associated with the perception of limbs movement (Restuccia et al., 2003; Seiss et al., 2003). Electrophysiological studies investigated spatiotemporal dynamics of reach-related neural activity for visual targets (Bernier et al., 2009) but, in contrast, similar attention was not given to understanding the neural processes underlying matching movements toward targets when visual feedback is absent and proprioception remains the only feedback available. Furthermore, the association between the brain response and the actual acuity at the joints still presents gaps to be filled. Concerning lower limbs, electrophysiological studies found that a performance worsening with age in a passive response time task is associated with a weaker and delayed proprioceptive afferent inflow to the cortex (Toledo et al., 2016) and a higher postural sway occurs in conjunction with stronger corticokinematic coherence, which reflects a poorer cortical proprioceptive processing and fewer proprioceptive afferences (Piitulainen et al., 2018). Investigating upper limb position sense, Ingemanson et al. (2019) observed that finger matching error in completely passive tasks correlates negatively with the connectivity between the ipsilesional primary motor cortex and secondary somatosensory cortex in individuals with chronic unilateral stroke (Ingemanson et al., 2019). Additionally, following stroke, decreased bimanual wrist position sense was suggested to be associated with decreased supramarginal gyrus functionality (Ben-Shabat et al., 2015), although no correlation was evaluated. These latter two works employed imaging techniques (fMRI) to investigate the neural correlates of upper-limb position sense. However, an electrophysiological investigation of the neural basis of position matching is missing, and few works have identified a link between brain activation and proprioceptive acuity level at the upper-limb joints.

Therefore, we were motivated to develop a protocol that combines electroencephalographic (EEG) measures and a robot-based quantitative test of proximal upper limb proprioception, to answer the following question: how does neural activity during a position sense test relate to proprioceptive acuity at the joints? We focused the attention on the μ frequency band (from 8 to 12 Hz), whose rhythm decreases, or desynchronizes, during motion (Pfurtscheller and Aranibar, 1979; Niedermeyer, 1997; McFarland et al., 2000). In particular, we investigated if and how μ activity at the offset of movement is associated with proprioceptive acuity during active and passive movements in a position matching task.

## 2. Materials and methods

### 2.1. Participants and experimental setup

**FIGURE 1:**
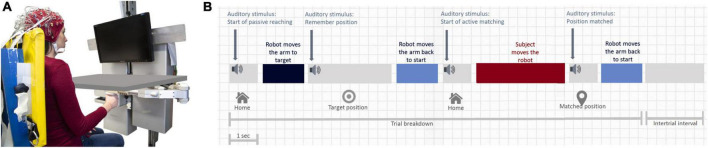
Experimental setup and protocol. **(A)** Robotic device with a board occluding vision of the arm. **(B)** Timeline of an experimental trial, consisting of passive matching, holding time, the first passive return, active matching, and final passive return. The duration of the active matching phase corresponds to the average duration employed by our sample of subjects (1.2 s from onset to offset + 2 s after the offset to let the subjects end completely his movement). The duration of the passive displacement toward each target was 1 s.

Eighteen participants (mean age ± std: 25.1 ± 4.2 years, 9 males, 9 females) with no history of motor and sensory disorders participated in the study after confirmation of their right-handedness through the Edinburgh Handedness Inventory (Oldfield, 1971). Subjects gave their informed consent to participate in the experiment, which was carried out in accordance with the Declaration of Helsinki, and it was approved by the local ethical committee of Liguria Region (no. 222REG2015, “Upper limb sensorimotor learning studies using robotic interfaces”). The experiment was carried out at the Motor Learning, Assistive and Rehabilitation Robotics Lab of the Istituto Italiano di Tecnologia (Genoa, Italy). The robotic device used in the experiments consists of a haptic, planar manipulandum with a large planar workspace (80 cm × 40 cm ellipse) and 2 degrees of freedom (Casadio et al., 2006). Two direct-drive brushless motors provide full back-drivability and low intrinsic mechanical impedance, and generate continuous forces ranging from 1 to 50 N. The device is also equipped with encoders to measure instantaneous hand position with high resolution (<0.1 mm). Subjects sat on an adjustable chair in front of the computer monitor (Figure 1A) and grasped the handle of the robot with their dominant right hand.

### 2.2. Experimental protocol

An ipsilateral position matching task, commonly used and widely recognized to measure position sense (Goble, 2010), was implemented on the robotic device, and brain activity was recorded through an EEG system during the whole test. The test consists of a series of center-out matching movements on a horizontal (transverse) plane at shoulder level and it has the overall goal of actively repeating as accurately as possible proprioceptive target positions, passively shown by the robot. During the test, a black board occluded vision of the arm such as subjects never saw their hand moving (Figure 1A). Figure 1B shows the four phases that characterize the timeline of each trial: (1) passive displacement of the hand by the robot, from the starting position (set in the midline at a distance of ∼20 cm from the chest) to a target position (*passive matching*); (2) holding time (3 s) during which subjects had to memorize the position of their hand, followed by the passive return to the reference starting position; (3) *active matching* movement performed by the subject with no assistance from the device, aimed at repeating the memorized position. The final phase (4) involved the passive movement back to the starting position. Phase (4) was folllowed by a 2-s-long intertrial interval. Passive displacements were implemented with a smooth, minimum jerk force profile. To guide subjects through the different phases, there were four auditory cues: at the beginning of the passive movement toward the target sounded a first cue (1024 Hz frequency beep lasting 45 ms) and, at its end, another cue signaled that the target had been reached (512 Hz, 45 ms). Subsequently, a third cue sounded to indicate that the active matching movement could begin (1024 Hz, 45 ms) and, finally, the last cue sounded when the subject reached the target position (512 Hz, 45 ms). Five different proprioceptive targets appeared randomly to avoid learning and replication of the same target every trial. These targets were on the arc of a circle at a 25 cm distance from the reference, and were located at −5, −2.5, 0, +2.5, and +5° with respect to the straight central line splitting vertically the monitor.

To investigate the effect of proprioceptive and visual feedback, we designed two conditions: *visuo-proprioceptive movement* (VPM) and *proprioceptive movement* (PM). In the VPM condition, for the entire duration of the trial, subjects were provided with visual feedback of their hand position as a yellow circular cursor of 1 cm moving on the screen. However, visual information about the target position was never displayed. For the sake of clarity, it is worth underlying that, in this condition, subjects could rely on and had to integrate both the visual (coming from the screen) and the proprioceptive information (coming from the moving body), which therefore represented two continuous sensory feedback channels. On the other hand, in the PM condition, no cursor was shown on the screen, so the subjects did not receive in any moment any visual feedback. In this case, subjects received specific guidelines to completely rely on and pay attention to the proprioceptive feedback coming from their arm and hand. Subjects performed 360 trials lasting about 2 h. In particular, the protocol comprised 6 target sets of 30 trials for each one of the two conditions. The two conditions were randomized among target sets and participants. Subjects also had the chance to rest and refocus between target sets. To avoid recalibration and continuous adjustments, subjects did not receive any feedback about their performance in the task.

### 2.3. Electrophysiological data recording and analysis

We applied the same procedure presented in our past study (Marini et al., 2019). EEG was acquired (sampling frequency: 2048 Hz) with a 64 active electrodes BioSemi ActiveTwo system (BioSemi B.V. Amsterdam), keeping electrode offsets below 35 mV and with a first-order analog anti-aliasing filter with a half-power cutoff at 3.6 kHz. The data were resampled at 512 Hz (bandwidth: 134 Hz), applying a 5th-order Hamming windowed sinc finite impulse response filter. Further electrodes were placed for electrooculography (EOG) recording. EEG data were processed within EEGLAB software (Delorme and Makeig, 2004). Data were resampled to 256 Hz and (0.1, 100 Hz) bandpass filtered. Through visual inspection, we identified and rejected segments with artifacts affected by subjects’ movement. To find artifacts we considered topographical/spectral distribution, as well as time series of the independent components extracted by EEGLAB ICA algorithm. Data were re-referenced to the common average reference and then segmented in (-600, 3000 ms) epochs around the event (time = 0).

The movement’s offset was the event around which we were interested in characterizing the subject’s response, in both the passive and the active matching, as they include target presentation and matching, respectively. The movement’s offset was set as the first instant at which the speed of the hand reached a value below 2 cm/s. Only when analyzing active motion, the movement’s offset was set as the first instant of a 2-s-long interval during which the speed of the hand was kept below the 2 cm/s.

For each epoch, we computed the event-related spectral perturbations (ERSPs) by applying Fast Fourier Transform (FFT) to 256 ms long (64 samples) overlapping time windows (shifting step: 8 ms) and a 16 points zero-padding and a Hanning-window tapering to increase smoothness and to reduce edge effects. Given its potential to catch both stimulus-evoked and -induced activity (Makeig, 1993; David et al., 2006), ERSPs have been chosen as measure to capture the brain activity as a function of time and frequency, around the offset of the movement. We computed the baseline spectral activity in a clean 500 ms-long window, starting from 1.1 s before the beginning of the trial. We adjusted individual epochs ERSPs (dB) with respect to the specified baseline (Makeig, 1993), after averaging the spectral estimate of multiple trials. The averaging process was performed across epochs for each condition, considering a (−400, 400 ms) time window around the *offset* and a (4, 32 Hz) frequency span.

**FIGURE 2:**
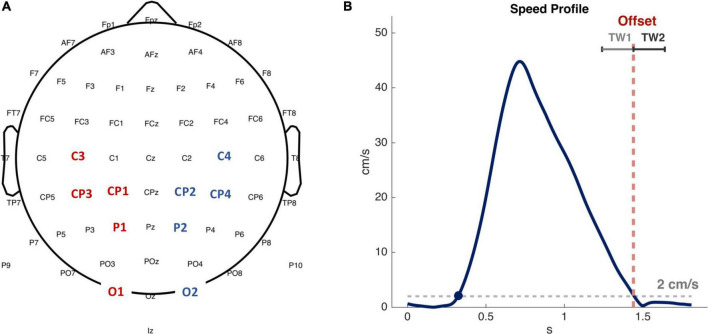
Electroencephalographic (EEG) analyzed ROIs and time intervals. **(A)** Channels considered in the analysis. Red and blue labels indicate channels in the left and right hemispheres, respectively. **(B)** An illustrative example of a bell-shaped speed profile corresponding to an active matching movement. The average duration of the active matching movement from the onset (blue dot) to the offset was 1.2 s for our sample of subjects. Red dotted line highlights movement offset, corresponding to the time in which the speed decreases below the threshold of 2 cm/s. TW1 and TW2 are the time windows of 200 ms in which EEG signals have been averaged and analyzed.

The regions of interest (ROIs) analyzed are represented in Figure 2: C3 and C4 electrodes, which are associated with *hand motor area* (Pfurtscheller et al., 1997; Burle et al., 2008), lCP (CP1 and CP3 electrodes on the left side) and rCP (CP2 and CP4 electrodes on the right side) associated with *sensorimotor area* (Christensen et al., 2007), P1 and P2 electrodes reflecting *association area* (Herwig et al., 2003), O1 and O2 electrodes linked to *visual area* (Neuper et al., 2005). We analyzed EEG activity of passive and active movements, within the time window preceding and following the offset: TW1: (−200, 0 ms) and TW2: (0, 200 ms), with 0 ms corresponding to the movement offset (see Figure 2). We considered the (8, 12 Hz) frequency band whose power decreases, or desynchronizes, during movements (Pfurtscheller and Aranibar, 1979; Niedermeyer, 1997; McFarland et al., 2000). As a note to the reader, we acknowledge that the (8, 12 Hz) frequency band can be found in literature labeled as either *α band* or μ *band*. We have chosen to refer to it throughout the manuscript as μ *band*. Given the time-frequency spectra, we firstly averaged the power over frequency bins in the μ *band.* Then, the resulting time course of power was averaged over time in each time windows (TW1 and TW2) to obtain the outcome measures of power on which we performed the statistical analysis.

### 2.4. Behavioral data analysis

Behavioral data were processed using MATLAB R2019b (Mathworks Inc., Natick, MS, USA). To describe subjects’ ability to accurately reproduce the target position, we chose two indicators: the *Matching error*, *ME*, and the *Variability*, *V*. We separately considered the two planar components of each indicator, the frontal (x) and sagittal (y). The *Matching error* is a measure of accuracy and it is computed as the absolute difference between the proprioceptive target position (end of the *passive matching*) and the hand position at the end of the *active matching* (Marini et al., 2016):


(1) $$M\,E_{x}=\frac{\sum_{i=1:N}\left|x_{i}-x_{T\,G}\right|}{N}\;$$

(2) $$M\,E_{y}=\frac{\sum_{i=1:N}\left|y_{i}-y_{T\,G}\right|}{N}$$

where *x_i_* and *y_i_* are the *x* and *y* coordinates of the hand’s final position at the end of the *active matching* in the *i*-trial, and *x*_*TG*_ and *y*_*TG*_ are the *x* and *y* coordinates of the ideal target position; *N* are the repetitions in each condition (*N* = 180).

On the other hand, the *Variability* represents the consistency, therefore the precision of subjects’ performance. For each condition, it is computed as the standard deviation, across the *N* trials, of the hand’s position at the end of the *active matching* (Marini et al., 2016):


(3) $$V_{x}=S\,t\,D\,\left(x_{i=1:N}\right)$$

(4) $$V_{y}=S\,t\,D\,\left(y_{i=1:N}\right)$$

### 2.5. Statistical analysis

The statistical analysis aimed at investigating the presence of a relation between the activation of specific brain areas during the task and proprioceptive acuity at the joints. Additionally, to better understand the meaning of this first result, we compared the level of activation of the four ROIs involved with each other. Finally, we analyzed the behavioral outcomes.

For these purposes, the first part of the analysis regarded the linear regression between behavioral indicators of performance and EEG data. The regression equation is:


(5) p=a⁢q+b

where *p* represents behavioral outcomes and *q* ERSP values. Notably, we were not interested in the slope (*a*) and the intercept (*b*) of the linear regression, but only in the strength of the linear relationship (*R*^2^). Particularly, we used linear regression to test the association of ERSP values (the average magnitude of μ desynchronization over time) with *ME* and *V* (averaged among trials), separately considering x and y directions. Positive correlations mean that larger activations (μ desynchronization) reduce *ME* or *V*. Significant correlations (*p* < 0.05) were corrected with Bonferroni’s, since each variable was tested multiple times [8 ROIs × 2 time-windows × 2 movements (active/passive)], considering different variables as predictors. Then, to understand deeply how different brain areas were involved in the task, we performed a statistical analysis on ERSP values in the four ROIs of the two hemispheres, during active and passive movements, and in the VPM and PM conditions. In this second part of the analysis, for each condition (VPM and PM) and phase (active and passive) independently, we performed a two-way ANOVA to investigate the ROI and hemisphere effects. Parametric statistical tests were chosen after verifying the normality of data by Shapiro-Wilk normality tests. Interactions, when significant, were followed by *post-hoc* paired *t*-tests, retaining results as significant when Bonferroni’s corrected *p* values were lower than 0.05. Finally, outcome behavioral data (*ME* and *V*) have been investigated in the two conditions: we performed two separate ANOVAs, respectively, considering *ME* and *V* as dependent variables, with factors condition (VPM and PM) and direction (x, y). *Post-hoc* two-tails paired *t*-tests further investigated significant effects, retaining results as significant when Bonferroni’s corrected *p*-values were lower than 0.05. All the *post hoc* comparisons are reported in Supplementary Table 1.

## 3. Results

The most relevant contribution of this study lies in the results of the correlation analysis, which investigates the link between brain activation and proprioceptive acuity. To fully comprehend the relevance of those results, we will then analyze in detail the EEG and behavioral results. We will first report the activation in the different ROIs and the two hemispheres, presenting the value of the ERSPs in dB at the offset of both the *passive* and *active matching* movements, averaged in the time windows preceding and following the event (TW1 and TW2, respectively). Finally, we will look at the behavioral performance, through the analysis of the two above-described indicators of behavioral metrics.

### 3.1. Correlation between behavioral metrics and ERSP

The linear regression analysis can reveal any potential association between the ERSP in the ROIs analyzed during active and passive movements and the value of the behavioral performance indicators, *ME*_*x*_, *ME*_*y*_, *V_*x*_*, and *V_*y*_*, averaged across trails. Positive correlations would mean that a greater μ desynchronization (corresponding to a stronger activation, i.e., higher absolute values of ERSP) is associated with higher proprioceptive acuity (lower *Matching error* or *Variability*).

**FIGURE 3:**
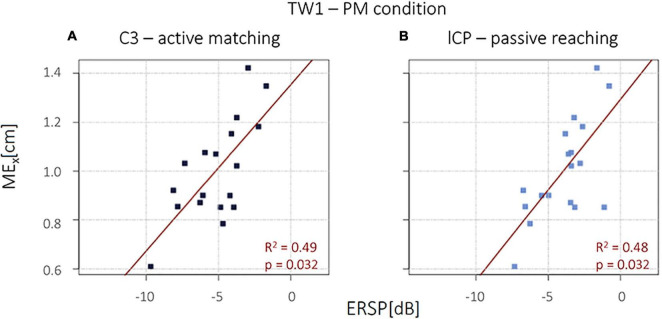
**(A)** Event-related spectral perturbation (ERSP) mean values in C3 during active movement. **(B)** ERSP mean values in lCP during passive movement.

Interestingly, in the PM condition, a significant positive correlation emerged between the magnitude of the μ desynchronization (ERSP) during the active movement in TW1 in the left central area C3 and the *ME*_*x*_ (*R^2^* = 0.49, *p* = 0.032), and between the *ME*_*x*_ and the μ desynchronization (ERSP) during the passive movement in TW1 in the left central parietal area lCP (*R^2^* = 0.48, *p* = 0.032), as shown in Figure 3. No other significant correlations were detected.

### 3.2. Differences among ROIs and between hemispheres

**FIGURE 4:**
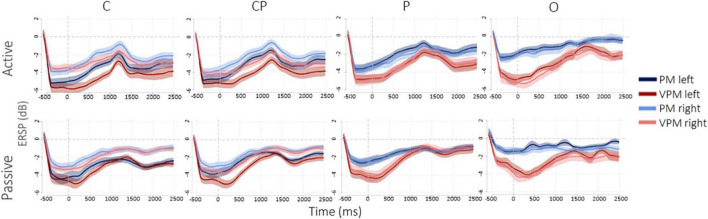
μ desynchronization in dB, from 500 ms before to 2500 ms after movement’s offset.

**FIGURE 5:**
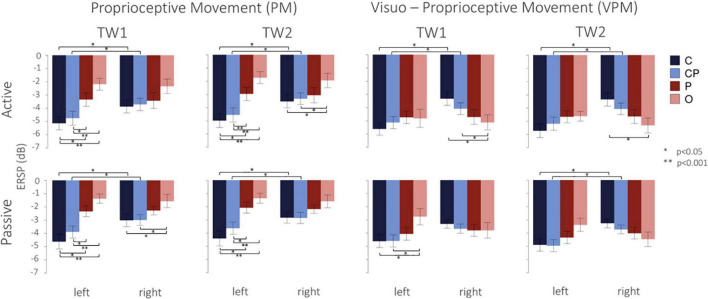
Mean and standard errors of ERSP values (dB) in the two conditions (PM and VPM) during active and passive movements (**top** and **bottom** line). Different colors indicate the four ROIs analyzed in the left and right hemispheres. Indicate a significant (*p < 0.05) and highly significant (**p < 0.001) difference, respectively, after Bonferroni’s correction.

Figure 4 shows the desynchronization in dB, from −500 ms before to 2500 ms after the movement offset. Overall, in the left hemisphere, C3 and lCP showed higher desynchronization, while, conversely, in the right hemisphere the desynchronization was more homogeneous and similar among the ROIs. As expected, desynchronization in O (both O1 and O2) and P (both P1 and P2) was higher in the visuo-proprioceptive condition, while in C3 and lCP did not consistently change for different conditions. Passive movements generally showed a less pronounced desynchronization in all the four ROIs and two conditions. Figure 5 presents the ERSP values in each ROI in the different conditions in TW1 and TW2, while Supplementary Figure 1 shows ERSPs of the whole scalp. Statistical results of the *post-hoc* tests are reported in the following subsections and in Supplementary Table 1.

#### 3.2.1. Active matching

In the proprioceptive movement condition (PM), a significant interaction was found between hemisphere and ROIs in both TW1 [*F*_(3,51)_ = 4.61, *p* = 0.006] and TW2 [*F*_(3,51)_ = 6.15, *p* = 0.001]. In the left hemisphere, we observed a desynchronization decreasing from C to CP to P and finally to O, with similar patterns in both time windows. *Post-hoc* tests revealed a stronger desynchronization in C than in O and P, and in CP than in O and P, with the largest differences found between C and O and between CP and O. Vice versa, in the right hemisphere, all the four ROIs showed a generally weaker desynchronization (Figure 5), with less pronounced differences among them: the only significant differences were a stronger desynchronization in C than O and in CP than O in TW2. In the visuo-proprioceptive movement condition (VPM), a significant interaction was found between hemisphere and ROIs in both TW1 [*F*_(3,51)_ = 11.59, *p* < 0.001] and TW2 [*F*_(3,51)_ = 19.74, *p* < 0.001]. In the left hemisphere, we observed similar results for both the time windows, indicating a similar strong desynchronization in all four ROIs (Figure 5). This was confirmed by the *post-hoc* tests revealing the absence of any significant difference (Supplementary Table 1). Vice versa, in the right hemisphere, a slight trend of increasing desynchronization from C to CP to P and finally to O appeared in both the time windows (Figure 5). The *post-hoc* tests revealed only a significantly higher desynchronization in O than in C and CP in TW1, and only between O and C in TW2 (Supplementary Table 1). Importantly, as regards the difference between the left and right hemispheres, in both conditions we found a stronger desynchronization in the left hemisphere only in C (TW1, PM: *t*_17_ = −3.24, *p* = 0.019; TW1,VPM: *t*_17_ = −5.19, *p* < 0.001; TW2, PM: *t*_17_ = −3.62, *p* = 0.008; TW2,VPM: *t*_17_ = −5.35, *p* < 0.001) and CP (TW1, PM: *t*_17_ = −3.78, *p* = 0.006; TW1,VPM: *t*_17_ = −3.03, *p* = 0.030; TW2, PM: *t*_17_ = −5.08, *p* < 0.001; TW2,VPM: *t*_17_ = −3.23, *p* = 0.020) during TW1 and TW2, while desynchronization in O and P did not differ between left and right.

#### 3.2.2. Passive matching

In the proprioceptive movement condition (PM), a significant interaction was found between hemispheres and ROIs in both TW1 [*F*_(3,51)_ = 9.21, *p* < 0.001] and TW2 [*F*_(3,51)_ = 8.79, *p* < 0.001]. *Post-hoc* tests revealed a significantly stronger desynchronization in C than in O and P, and in CP than in O and P in the left hemisphere, with a decreasing trend of desynchronization starting from C to CP to P and finally to O in both time windows (Figure 5). In the right hemisphere, the only significant difference was a lower desynchronization in O with respect to C and CP during TW1 (Supplementary Table 1). In the visuo-proprioceptive movement condition (VPM), a significant interaction was found between hemisphere and condition in both TW1 [*F*_(3,51)_ = 7.33, *p* < 0.001] and TW2 [*F*_(3,51)_ = 12.78, *p* < 0.001]. In the left hemisphere, the *post-hoc* tests revealed a significant difference between O and both C and CP only n TW1, with a lower desynchronization in O (Supplementary Table 1). In the right hemisphere, we found similar results for both time windows, indicating a comparable desynchronization in all four ROIs (Figure 5). This was confirmed by the *post-hoc* tests revealing no significant difference in the level of desynchronization in the ROIs, neither in TW1 nor in TW2 (Supplementary Table 1). As regards the difference between the left and right hemispheres, for both the conditions, in TW2, we found a stronger desynchronization in the left hemisphere only in C (PM: *t*_17_ = −3.59, *p* = 0.009; VPM: *t*_17_ = −3.32, *p* = 0.016) and CP (PM: *t*_17_ = −2.86, *p* = 0.043; VPM: *t*_17_ = −3.45, *p* = 0.012), while desynchronization in O and P did not differ between left and right (Figure 5). Analog results were observed in TW1 for the PM condition (C: *t*_17_ = −3.74, *p* = 0.007; CP: *t*_17_ = −3.24, *p* = 0.019), while no differences were found in the VPM condition in TW1 between the left and right hemispheres in none of the ROIs.

### 3.3. Behavioral results

**FIGURE 6:**
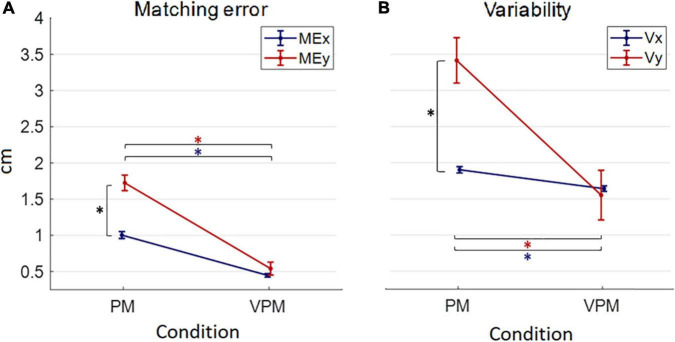
Mean and standard errors of behavioral performance indicators (matching error ME in panel **A** and variability V in panel **B**) in the two conditions (proprioceptive PM and visuo-proprioceptive VPM). The blue or red “*” indicates a significant difference (*p* < 0.05) between PM and VPM conditions along each direction. The black “*” points out a significant difference between the behavioral indicators along the direction x and y in each condition.

Generally, in the PM condition, both *Matching error* and *Variability* resulted higher in the sagittal (*ME_*y*_, V_*y*_*) than in the frontal direction (*ME_*x*_, V_*x*_*); in contrast, direction appeared to be irrelevant when visual feedback was added (Figure 6). Moreover, unsurprisingly, *Matching error* and *Variability* resulted lower in the VPM condition (Figure 6).

As for the *Matching error*, statistical analysis revealed a significant interaction between direction and condition [*F*_(1,17)_ = 21.09, *p* < 0.001]. *Post hoc* analysis found a significant difference between PM and VPM for both *ME*_*x*_ and *ME*_*y*_ with a lower error in the VPM condition (*ME*_*x*_: *t*_17_ = 11.09, *p* < 0.001, *ME*_*y*_: *t*_17_ = 7.94, *p* < 0.001). A similarly, significant interaction was found for the *Variability* [*F*_(1,17)_ = 13.30, *p* = 0.001] with the *post-hoc* test confirming results emerged for the *Matching error*: precisely, it highlighted a significant difference between PM and VPM for both *V*_*x*_ and *V*_*y*_ with a lower *Variability* in the VPM condition (*V*_*x*_: *t*_17_ = 5.38, *p* < 0.001; *V*_*y*_: *t*_17_ = 4.29, *p* < 0.001).

Regarding the difference between errors along the frontal, x, and sagittal direction, y, an interesting similar pattern of behavior emerged for *Matching error* and *Variability*. In particular, the direction influenced the magnitude of the error only in the PM condition, while errors along x and y were not significantly different in the VPM condition (Figure 6). Indeed, the difference between *x* and *y* axis was highly significant for the *Matching error* and the *Variability* in the PM condition (*ME*: *t*_17_ = −7.46, *p* < 0.001; *V_:_ t*_17_ = 4.70, *p* < 0.001) and reached significance for neither *ME* nor *V* in the VPM condition (*ME*: *t*_17_ = −1.06, *p* = 0.061; *V_:_ t*_17_ = 0.25, *p* = 1.000) (Figure 6).

## 4. Discussion

Generally speaking, proprioception plays a key role in the motor performances of our daily living activities, particularly in relation to the gestures of the upper limb that are crucial for purposive actions. The present study is the first one trying to specifically correlate upper limb position sense with neural activity; in other words, this is the first attempt to clarify to what extent a link exists between brain activation and the level of proprioceptive acuity at the joints. The main finding of this study indicates a significant correlation between upper limb sensory acuity and activation in the left central and central-parietal brain regions. Further analysis performed revealed a high involvement of these same regions during the processing of information related to the joint position. Overall, the experimental protocol designed allowed the collection of a broad set of experimental data that lead to significative behavioral and electrophysiological results. The structure of the protocol allow us to compare the results obtained under different conditions (VPM and PM) and phases of the task (passive or active matching movements), with the aim of improve the present knowledge about of proprioceptive processing.

As regards behavioral results, as expected, we found lower *ME* and *V* when visual feedback was provided. Interestingly, we found higher errors along the sagittal (y) than the frontal direction (x), but this difference occurred only in the PM condition. From these results, we hypothesized that when subjects rely only on proprioceptive feedback, they do not compensate for human arm viscoelastic properties that make elbow flexion/extension easier to perform than shoulder flexion/extension (Frolov et al., 2006). Contrarily, when visual feedback was available, subjects were able to correct their behavior, leading the movement back to isotropy.

Concerning EEG data, these results offer a characterization of the neural correlates of upper limb position sense, in terms of lateralization between the two hemispheres and comparison among the ROIs. As regards the difference between the two hemispheres, the left one exhibited a higher activation level in the motor and somatosensory areas (C3 and lCP, respectively), which, being contralateral to the moving limb, represent indeed those areas mostly involved in movement execution (Luft et al., 2002; Carson, 2005) and kinesthetic perception of limb motion (Naito et al., 2005, 2007). Conversely, such higher activation levels in the left hemisphere did not occur in the occipital or the parietal area (respectively, considered to identify association and visual regions). More in general, when considering both hemispheres, we discovered that the four cortical areas presented different levels of activation under the two feedback conditions. Indeed, in the PM condition, we found higher activation in the central and central-parietal areas, rather than parietal and occipital. In the VPM condition, the contribution of P and O consistently increased, but the high activation of C and CP remained, thus confirming the contribution of motor and somatosensory areas for the processing arm position sense and kinesthesia. The activation of both C and CP remained comparable regardless of whether the visual information was available. Although healthy subjects are known to rely generally more on visual than on proprioceptive information (Mon-Williams et al., 1997), these findings remark that, during movement, afferent proprioceptive information is processed in the same way either in presence or absence of vision.

Finally, we consider the results of the linear regression analysis the most interesting and relevant of this work. Remarkably, we observed a significant positive correlation between the strength of the activation during the active movements in the left central area C3 and the *ME*_*x*_ and between the *ME*_*x*_ and the μ desynchronization during the passive movement in the left central-parietal area lCP. Both positive correlations were found in the time window preceding the event, TW1. No other significant correlation emerged. These results provide evidence of a direct link between the level of activation in the brain and limb proprioceptive acuity.

Indeed, it is certainly interesting that a significant correlation emerged between the error representing proprioceptive acuity (*ME*) and the activation of the cortical areas responsible for motor planning and execution and sensorimotor processing (Luft et al., 2002; Carson, 2005; Naito et al., 2005). Further support for the significance of such correlations lies in the fact that no other significant correlation emerged in P and O. There are some more aspects of this finding worth discussing: firstly, we found significant correlations only in the PM condition and not in the VPM, while we could have expected a relationship with the *ME* or V and the activation in the occipital area, responsible for visual processing (Neuper et al., 2005), or in P, as in the VPM condition subjects had to integrate both visual and proprioceptive feedback (Masterton and Berkley, 1974; Culham and Kanwisher, 2001). A possible explanation for the presence of a correlation only in the PM condition lies in the saturation of activation that emerged in the four ROIs during the VPM condition, which seems to reflect the saturation of information reached by providing visual feedback. We may speculate that, in the VPM case, subjects were in a condition of feedback redundancy which led to a conflict between sensory afferences (Balslev et al., 2005; Judkins and Scheidt, 2014). Indeed, both the proprioceptive and the visual feedback related to the same information about the position of the end effector. However, their reference systems were different (joint/internal vs. world/external) and the visual modality is known to dominate over proprioception when planning movements (Sarlegna and Sainburg, 2009). For this reason, we hypothesize that their integration may lead to a sort of competition or conflict for state estimation (Judkins and Scheidt, 2014). Additionally, significant correlations were found only in the frontal direction (*ME*_*x*_). We could identify the higher variability that emerged in the sagittal direction (y) as the reason preventing the appearance of interesting correlations with *ME_*y*_.* Indeed, this higher variability reflects the noise that affects the nervous system, from the generation of sensory responses to the development of motor commands, which is a crucial problem for the correct processing of afferent information (Faisal et al., 2008). Even if the central nervous system has an impressive ability to minimize the sensory uncertainty and movement variability coming from such internal noise (Bays and Wolpert, 2007), it is evident that the more inaccurate and variable (noisy) the input sensory signals are, the greater the uncertainty in the estimation of the state (Bard et al., 1995).

Furthermore, it is not surprising that significant correlations involved only the *ME*, which represents subjects’ accuracy and the asymptotic value that would result from an infinite number of trials. On the other side, the *Variability* does not depend on how well the subject encodes and then replicates the target but rather represents how consistently he/she repeats the same estimation. This replication is affected by several factors, which may lead to higher kinematic dispersion. Finally, the linear relationship emerged only in TW1: this agrees with the hypothesis formulated through the linear regression equation, namely, that is μ desynchronization to predict the following behavioral outcome. Indeed, the behavioral outcome is computed considering subjects’ position right after TW1, i.e., at the offset of movement, when the subject is passively stopped (passive matching phase) or decides to stop (active matching phase).

In future studies, it will be interesting to investigate if such results are consistent in other areas and directions of the workspace and with results emerging from other types of proprioceptive tests (Cappello et al., 2015). Therefore, other aspects of EEG data, such as the activation in the β band (see Supplementary Figures 2, 3), could be correlated with behavioral results to investigate a potential link between the magnitude of such effect and proprioceptive acuity (Haar et al., 2019). Additionally, the position matching task could be used in future to investigate the effects of an attentional load on μ desynchronization (Hobson and Bishop, 2016), such as what happens during the phases that follows the movement offsets here analyzed (i.e., “target position” and “matching position” phases) (Figure 1), when memory work is required. A deeper knowledge of the neural basis underlying proprioception will help understand the mechanisms that lead to its deterioration with age (Barresi et al., 2022) or in presence of neurological diseases (De Santis et al., 2015). Nonetheless, the method here proposed appears to be a possible general framework, which can be suitable for a comprehensive investigation of sensorimotor integration.

## 5. Conclusion

Sensory feedback regarding upper limb position is pivotal for proper movement execution in the surrounding environment. Furthermore, reduced proprioceptive acuity may lead to poor motor control and bodily perception, which are strongly linked to morbidity and mortality in older age. In the present study, we sought a link between the neural activity related to the upper limb position sense and the proprioceptive acuity in a position-matching task. A brain–behavior approach revealed that higher activity in motor (central) and sensorimotor (central-parietal) cortical areas contralateral to the moving limb in active and passive phases, respectively, is associated with the error representing proprioceptive acuity. Since no similar relationship was found in other brain areas, this evidence supports the existence of a specific link between brain areas responsible for upper limb proprioceptive processing and proprioceptive acuity at the joints.

## Data availability statement

The raw data supporting the conclusions of this article will be made available by the authors, without undue reservation.

## Ethics statement

The studies involving human participants were reviewed and approved by the Liguria Region (no. 222REG2015). The patients/participants provided their written informed consent to participate in this study.

## Author contributions

FM and JZ designed the study and formulated the experimental question. FM collected the data and performed the data analysis. GA and CC participated in the data analysis. GA and FM wrote the manuscript. JZ supervised the study. PM and JZ participated in results interpretation and manuscript revision. All authors approved the final version of the manuscript.
