# Supplementary material for: μ-band desynchronization in the contralateral central and central-parietal areas predicts proprioceptive acuity
Source: Front Hum Neurosci. 2023 Mar 15;17:1000832. doi: 10.3389/fnhum.2023.1000832 (PMC10050694; doi:10.3389/fnhum.2023.1000832)
Supplement: Supplementary file 1 [file Data_Sheet_1.docx]

Supplementary Material

# Supplementary Table

|  |  |  |  |  | **t** | **df** | **p** | **Cohen's d** |
| --- | --- | --- | --- | --- | --- | --- | --- | --- |
| **C-CP** | **active** | **PM** | **TW1** | **L** | -1.16 | 17 | 1.000 | -0.74 |
|  |  |  | **TW1** | **R** | -0.46 | 17 | 1.000 | -0.31 |
|  |  |  | **TW2** | **L** | -1.34 | 17 | 1.000 | -0.81 |
|  |  |  | **TW2** | **R** | -0.63 | 17 | 1.000 | -0.42 |
|  |  | **VPM** | **TW1** | **L** | -1.53 | 17 | 1.000 | -1.04 |
|  |  |  | **TW1** | **R** | 3.25 | 17 | 0.056 | 1.63 |
|  |  |  | **TW2** | **L** | -1.63 | 17 | 0.732 | -1.09 |
|  |  |  | **TW2** | **R** | 3.26 | 17 | 0.056 | 1.51 |
|  | **passive** | **PM** | **TW1** | **L** | -2.10 | 17 | 0.608 | -1.43 |
|  |  |  | **TW1** | **R** | -0.14 | 17 | 1.000 | -0.09 |
|  |  |  | **TW2** | **L** | -2.33 | 17 | 0.385 | -1.52 |
|  |  |  | **TW2** | **R** | 0.08 | 17 | 1.000 | 0.05 |
|  |  | **VPM** | **TW1** | **L** | -0.03 | 17 | 1.000 | -0.02 |
|  |  |  | **TW1** | **R** | 2.05 | 17 | 0.668 | 1.06 |
|  |  |  | **TW2** | **L** | 0.15 | 17 | 1.000 | 0.11 |
|  |  |  | **TW2** | **R** | 2.73 | 17 | 0.171 | 1.39 |
| **C-P** | **active** | **PM** | **TW1** | **L** | -3.70 | 17 | **0.021** | -3.55 |
|  |  |  | **TW1** | **R** | -0.71 | 17 | 1.000 | -0.82 |
|  |  |  | **TW2** | **L** | -4.34 | 17 | **0.005** | -3.93 |
|  |  |  | **TW2** | **R** | -0.76 | 17 | 1.000 | -0.87 |
|  |  | **VPM** | **TW1** | **L** | -2.01 | 17 | 0.725 | -1.87 |
|  |  |  | **TW1** | **R** | 3.01 | 17 | 0.095 | 2.64 |
|  |  |  | **TW2** | **L** | -2.36 | 17 | 0.365 | -2.23 |
|  |  |  | **TW2** | **R** | 2.89 | 17 | 0.121 | 2.51 |
|  | **passive** | **PM** | **TW1** | **L** | -4.43 | 17 | **0.004** | -4.73 |
|  |  |  | **TW1** | **R** | -1.69 | 17 | 1.000 | -1.84 |
|  |  |  | **TW2** | **L** | -4.54 | 17 | **0.003** | -4.73 |
|  |  |  | **TW2** | **R** | -1.70 | 17 | 1.000 | -1.71 |
|  |  | **VPM** | **TW1** | **L** | -1.44 | 17 | 1.000 | -1.15 |
|  |  |  | **TW1** | **R** | 1.27 | 17 | 1.000 | 1.28 |
|  |  |  | **TW2** | **L** | -1.59 | 17 | 1.000 | -1.22 |
|  |  |  | **TW2** | **R** | 1.69 | 17 | 1.000 | 1.91 |
| **C-O** | **active** | **PM** | **TW1** | **L** | -7.06 | 17 | **0.000** | -6.20 |
|  |  |  | **TW1** | **R** | -3.08 | 17 | 0.081 | -2.96 |
|  |  |  | **TW2** | **L** | -7.53 | 17 | **0.000** | -6.66 |
|  |  |  | **TW2** | **R** | -3.36 | 17 | **0.045** | -3.03 |
|  |  | **VPM** | **TW1** | **L** | -0.99 | 17 | 1.000 | -1.37 |
|  |  |  | **TW1** | **R** | 2.50 | 17 | 0.274 | 3.35 |
|  |  |  | **TW2** | **L** | -1.92 | 17 | 0.856 | -2.48 |
|  |  |  | **TW2** | **R** | 2.79 | 17 | 0.151 | 3.68 |
|  | **passive** | **PM** | **TW1** | **L** | -6.93 | 17 | **0.000** | -6.99 |
|  |  |  | **TW1** | **R** | -3.51 | 17 | **0.032** | -3.03 |
|  |  |  | **TW2** | **L** | -6.16 | 17 | **0.000** | -6.42 |
|  |  |  | **TW2** | **R** | -3.17 | 17 | 0.068 | -2.70 |
|  |  | **VPM** | **TW1** | **L** | -3.35 | 17 | **0.045** | -3.33 |
|  |  |  | **TW1** | **R** | 0.75 | 17 | 1.000 | 1.01 |
|  |  |  | **TW2** | **L** | -3.09 | 17 | 0.080 | -3.14 |
|  |  |  | **TW2** | **R** | 2.02 | 17 | 0.712 | 2.67 |
| **CP-P** | **active** | **PM** | **TW1** | **L** | -4.11 | 17 | **0.009** | -2.72 |
|  |  |  | **TW1** | **R** | -0.61 | 17 | 1.000 | -0.56 |
|  |  |  | **TW2** | **L** | -4.96 | 17 | **0.001** | -3.04 |
|  |  |  | **TW2** | **R** | -0.55 | 17 | 1.000 | -0.53 |
|  |  | **VPM** | **TW1** | **L** | -1.08 | 17 | 1.000 | -0.83 |
|  |  |  | **TW1** | **R** | 1.59 | 17 | 1.000 | 1.23 |
|  |  |  | **TW2** | **L** | -1.44 | 17 | 1.000 | -1.10 |
|  |  |  | **TW2** | **R** | 1.49 | 17 | 1.000 | 1.19 |
|  | **passive** | **PM** | **TW1** | **L** | -4.76 | 17 | **0.002** | -3.65 |
|  |  |  | **TW1** | **R** | -2.51 | 17 | 0.268 | -1.92 |
|  |  |  | **TW2** | **L** | -4.77 | 17 | **0.002** | -3.55 |
|  |  |  | **TW2** | **R** | -2.48 | 17 | 0.284 | -1.77 |
|  |  | **VPM** | **TW1** | **L** | -1.51 | 17 | 1.000 | -1.16 |
|  |  |  | **TW1** | **R** | 0.45 | 17 | 1.000 | 0.35 |
|  |  |  | **TW2** | **L** | -1.81 | 17 | 1.000 | -1.34 |
|  |  |  | **TW2** | **R** | 0.82 | 17 | 1.000 | 0.74 |
| **CP-O** | **active** | **PM** | **TW1** | **L** | -5.86 | 17 | **0.000** | -5.25 |
|  |  |  | **TW1** | **R** | -3.28 | 17 | 0.053 | -2.75 |
|  |  |  | **TW2** | **L** | -6.45 | 17 | **0.000** | -5.68 |
|  |  |  | **TW2** | **R** | -3.33 | 17 | **0.048** | -2.77 |
|  |  | **VPM** | **TW1** | **L** | -0.40 | 17 | 1.000 | -0.54 |
|  |  |  | **TW1** | **R** | 1.68 | 17 | 1.000 | 2.01 |
|  |  |  | **TW2** | **L** | -0.98 | 17 | 1.000 | -1.28 |
|  |  |  | **TW2** | **R** | 2.09 | 17 | 0.622 | 2.48 |
|  | **passive** | **PM** | **TW1** | **L** | -8.64 | 17 | **0.000** | -6.21 |
|  |  |  | **TW1** | **R** | -3.66 | 17 | **0.023** | -3.15 |
|  |  |  | **TW2** | **L** | -6.45 | 17 | **0.000** | -5.43 |
|  |  |  | **TW2** | **R** | -2.83 | 17 | 0.140 | -2.75 |
|  |  | **VPM** | **TW1** | **L** | -3.45 | 17 | **0.036** | -3.37 |
|  |  |  | **TW1** | **R** | 0.23 | 17 | 1.000 | 0.28 |
|  |  |  | **TW2** | **L** | -3.18 | 17 | 0.066 | -3.28 |
|  |  |  | **TW2** | **R** | 1.39 | 17 | 1.000 | 1.65 |
| **P-O** | **active** | **PM** | **TW1** | **L** | -2.88 | 17 | 0.125 | -2.43 |
|  |  |  | **TW1** | **R** | -2.84 | 17 | 0.136 | -1.90 |
|  |  |  | **TW2** | **L** | -3.14 | 17 | 0.072 | -2.61 |
|  |  |  | **TW2** | **R** | -2.79 | 17 | 0.076 | -1.96 |
|  |  | **VPM** | **TW1** | **L** | 0.10 | 17 | 1.000 | 0.12 |
|  |  |  | **TW1** | **R** | 0.69 | 17 | 1.000 | 0.74 |
|  |  |  | **TW2** | **L** | -0.09 | 17 | 1.000 | -0.10 |
|  |  |  | **TW2** | **R** | 1.16 | 17 | 1.000 | 1.25 |
|  | **passive** | **PM** | **TW1** | **L** | -3.18 | 17 | 0.066 | -2.53 |
|  |  |  | **TW1** | **R** | -1.95 | 17 | 0.814 | -1.75 |
|  |  |  | **TW2** | **L** | -1.98 | 17 | 0.766 | -1.78 |
|  |  |  | **TW2** | **R** | -1.48 | 17 | 1.000 | -1.44 |
|  |  | **VPM** | **TW1** | **L** | -2.64 | 17 | 0.206 | -2.36 |
|  |  |  | **TW1** | **R** | -0.01 | 17 | 1.000 | -0.01 |
|  |  |  | **TW2** | **L** | -2.07 | 17 | 0.643 | -1.95 |
|  |  |  | **TW2** | **R** | 0.91 | 17 | 1.000 | 0.93 |

Supplementary Table 1: Bonferroni-corrected post-hoc comparisons between ROIs activations in the active/passive phase of movements and in the two time-windows (TW1/TW2). Significant comparisons (p < 0.05) are marked in red.


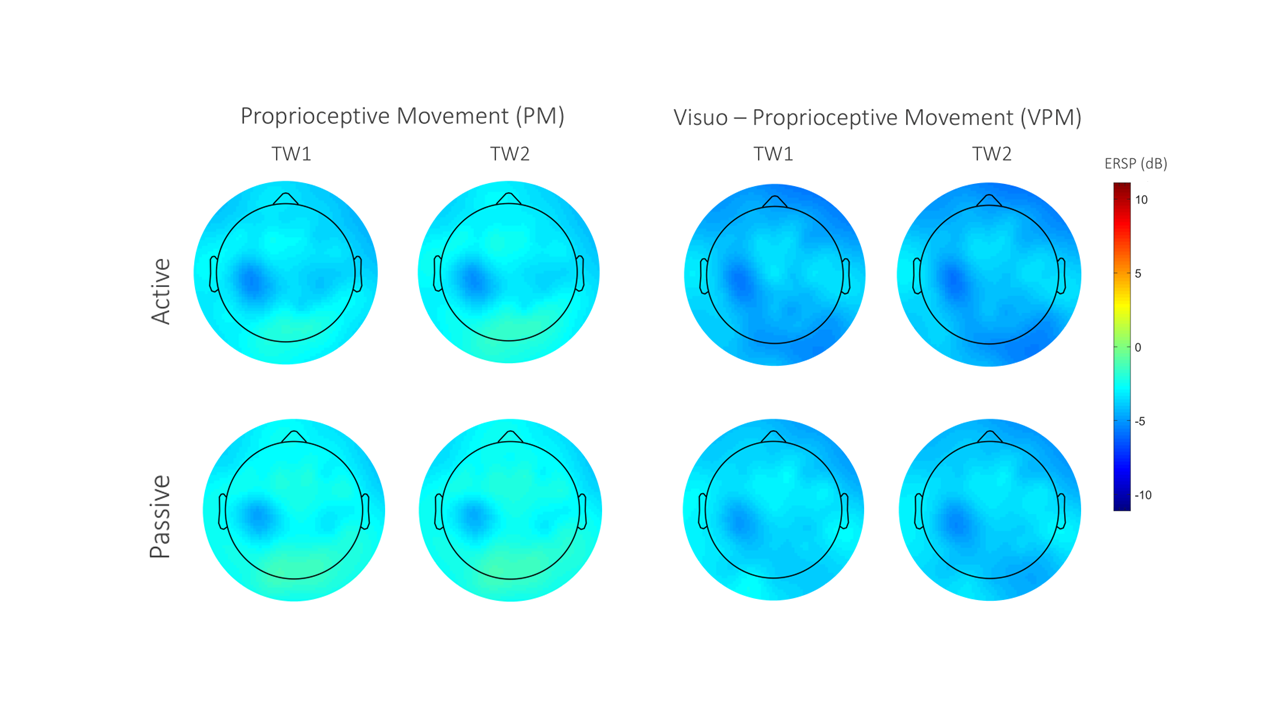


Supplementary Figure 1: Average µ band desynchronization in the whole scalp during TW1 and TW2, i.e. in the 200 ms interval preceding and following movement’s offset.


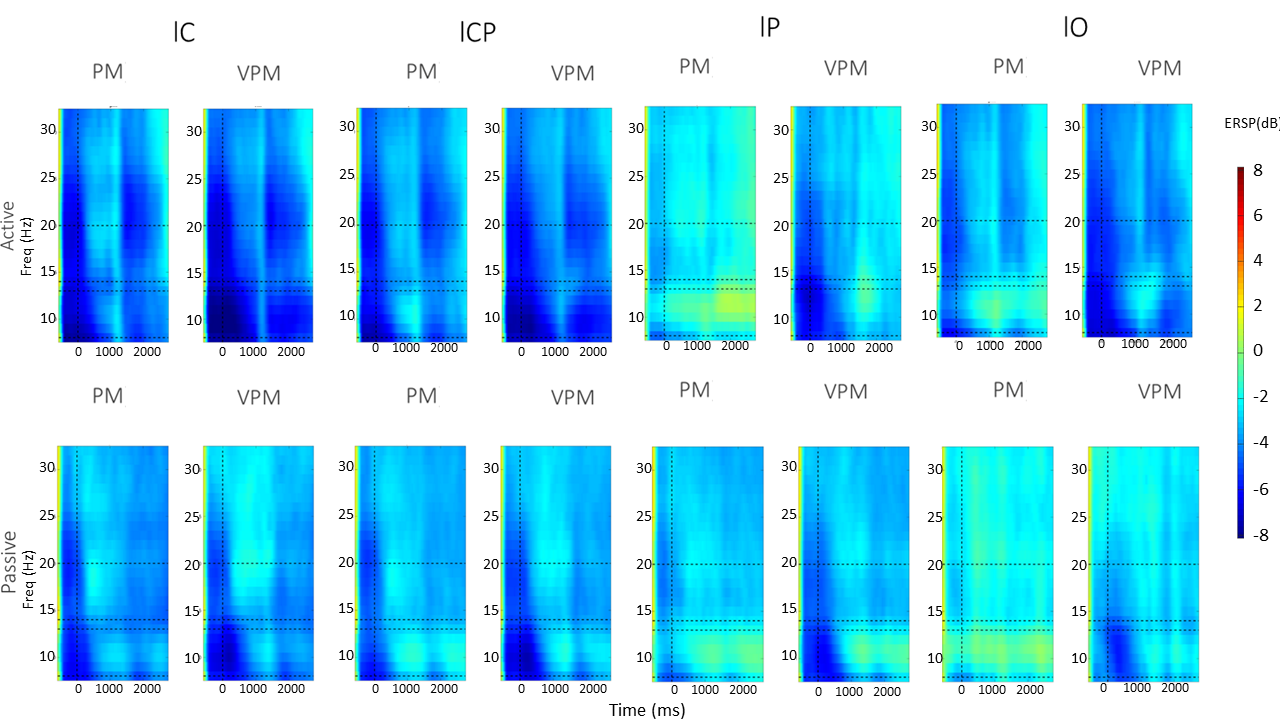


Supplementary Figure 2: Time-frequency spectra of the four ROIs in the left hemisphere. The interval of frequencies shown here is 8-20 Hz, which correspond to µ (8-12 Hz) and β1 (14-20 Hz).


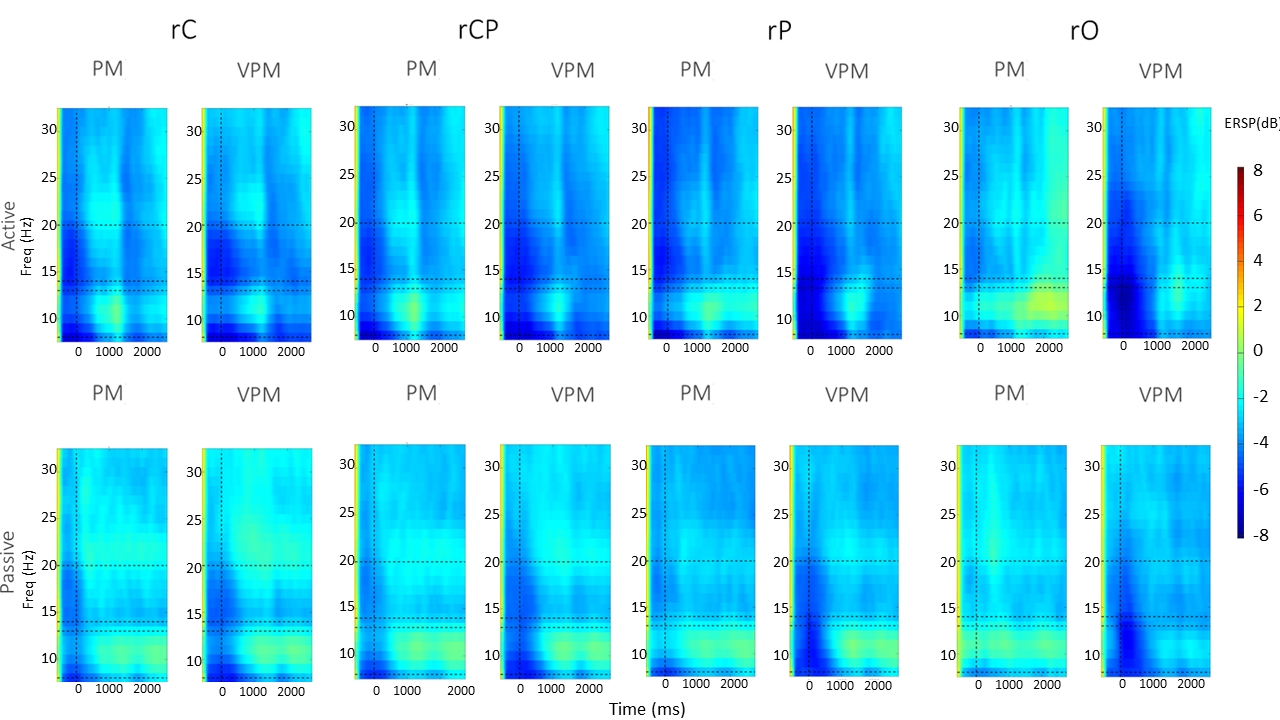


Supplementary Figure 3: Time-frequency spectra of the four ROIs in the rigth hemisphere. The interval of frequencies shown here is 8-20 Hz, which correspond to µ (8-12 Hz) and β1 (14-20 Hz).
